# New Alkaloid and Aromatic Glucoside from the Flowers of *Cymbidium* Lunagrad Eternal Green

**DOI:** 10.3390/molecules23010099

**Published:** 2018-01-03

**Authors:** Li-Yan Song, Fang Huang, Yan Wang, Zu-Jian Wu, Ming-An Ouyang

**Affiliations:** State Key Laboratory of Ecological Pest Control for Fujian and Taiwan Crops, Institute of Plant Virology, Fujian Agriculture and Forestry University, Fuzhou 350002, Fujian, China; slywinter@163.com (L.-Y.S.); fafuhf12@163.com (F.H.); fafuwy12@163.com (Y.W.); wuzujian@126.com (Z.-J.W.)

**Keywords:** orchid, *Cymbidium* Lunagrad Eternal Green, alkaloid, aromatic glucoside, flavone glycoside, natural product

## Abstract

In this paper, we investigated the chemical components of the flowers of *Cymbidium* Lunagrad Eternal Green for the first time. In the whole post-fertilization, a new alkaloid, named Lunagrad A (**1**), and a new aromatic glucoside, named Lunagrad B (**2**), were isolated from the MeOH extract of the flowers of *Cymbidium* Lunagrad Eternal Green, along with other six known aromatic compounds (**3**–**8**) and three flavone glucosides (**9**–**11**). These structures were determined on the basis of NMR experiments, as well as chemical evidence.

## 1. Introduction

Since ancient times, numerous orchid species have been used in different countries for apparent ornamental and medicinal value. *Cymbidium* hybridum is one of the most popular pot orchids worldwide, which contains a huge number of cultivars with large flowers. Commercial *Cymbidium* hybridum includes some wild species in genus of *Cymbidium* and lots of hybrid cultivars through inter-specific hybridization between 20 *Cymbidium* species of terrestrial and epiphytic orchids. The natural parent species of *Cymbidium* hybridum could trace back to its primal putative parents *Cymbidium eburneum* (maternal parent) and *Cymbidium lowianum* (paternal parent) in 1889 [1,2,3]. However, very little is known about the chemical or bioactive substances of these cultivars. To the best of our knowledge, only one *Cymbidium* hybridum, *Cymbidium* Great Flower Marylaurencin, has been chemically investigated, leading to the identification of phenanthrene derivatives and aromatic constituents, which showed certain biological activities [4,5,6,7].

**Figure 1:**
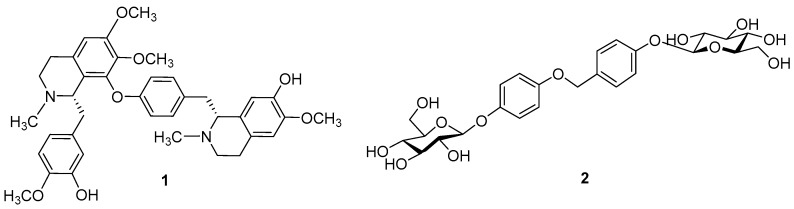
Two new compounds **1** and **2** isolated from the flowers of *Cymbidium* Lunagrad Eternal Green.

In this paper, we investigated the chemical components of the flowers of *Cymbidium* Lunagrad Eternal Green for the first time. In the whole post-fertilization, a new alkaloid, named Lunagrad A (**1**), and a new aromatic glucoside, named Lunagrad B (**2**), were isolated (Figure 1). We describe here the isolation, purification, and structural elucidation of **1** and **2**, primarily determined by extensive NMR experiments.

**Figure 2:**
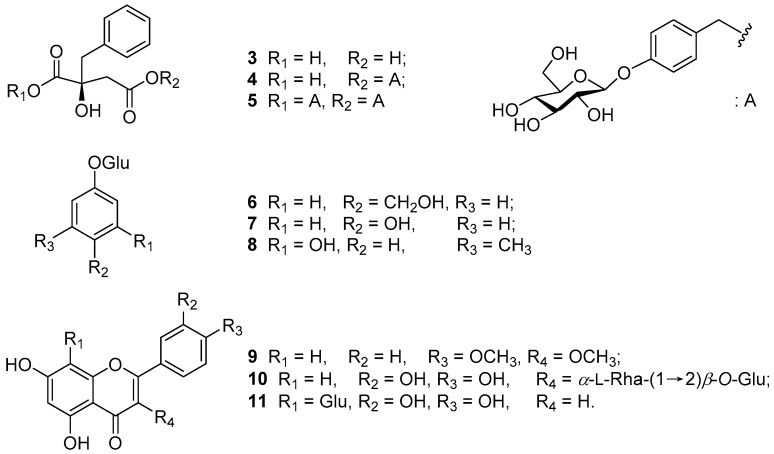
Nine known compounds **3**–**11** isolated from the flowers of *Cymbidium* Lunagrad Eternal Green.

In addition, six known aromatic compounds (2*R*)-2-benzyl-2-hydroxysuccinic acid (**3**) [8], marylaurecinosides B (**4**) [5], grammatophylloside B (**5**) [9], gastrodin (**6**) [10], arbutin (**7**) [11], and sakakin (**8**) [12], together with three flavone glucosides, ermanin (**9**) [13], quercetin 3-*O*-α-(2″-*O*-α-l-rhamnopyranosyl-β-d-glucopyranoside (**10**) [14], and orientin (**11**) [15] were found. The known structures (Figure 2) were identified by comparison of their spectroscopic data with those reported in the literature. It is noteworthy that compounds **3**–**8** were also obtained from the flowers of *Cymbidium* Great Flower Marylaurencin [7].

## 2. Results and Discussion

Lunagrad A (**1**) was identified as a bisbenzyltetrahydroisoquinoline alkaloid (BBIQA) derivative. The BBIQAs represent a large and important class of natural products, which have received great attention due to their wide range of pharmacological activities [16,17,18,19,20,21]. Compound **1** was obtained as a slightly yellow amorphous powder and showed an [M + H]^+^ ion peak at *m/z* = 641.3230 (calcd. 641.3227) in the HR-MS (ESI), which corresponded to the molecular formula C_38_H_44_N_2_O_7_. ^13^C-NMR, edited HSQC, and HMBC spectra (Supplementary Information Figures S3–S8) of compound **1** displayed 38 carbon resonances, assignable to ten aromatic tertiary carbons (δ_C_ 107.3, 113.2, 113.7, 116.7, 121.4, 122.8, 122.9, 124.4, 131.8, 133.9), 14 aromatic quaternary carbons (δ_C_ 124.0, 129.0, 129.1, 129.3, 135.8, 136.7, 139.3, 153.0, 149.4, 148.7, 151.0, 150.3, 145.2, 155.1), two aliphatic methines (δ_C_ 63.0, 64.5), six aliphatic methylenes (δ_C_ 23.7, 26.0, 37.5, 42.6, 45.3, 45.7), two NCH_3_ (δ_C_ 42.5, 43.1), and four OCH_3_ groups (δ_C_ 56.1, 56.4, 56.8, 60.7) that are connected to aromatic rings. The NMR data featured a 1,4,5-trisubstituted benzene moiety [δ_H_ 6.56 (s) H-13; δ 6.92, 6.86 (2H, both d, *J* = 8.2 Hz) H-16, H-17], and a 1,4-disubstituted benzene moiety [δ_H_ 7.43, 7.05 (2H, both d, *J* = 8.2 Hz) H-13′, H-14′; δ_H_ 6.76, 6.38 (2H, both d, *J* = 8.2 Hz) H-16′, H-17′], together with three aromatic proton singlets (δ_H_ 6.42, H-6; δ 6.66, H-6′; δ 6.01, H-9′) [21]. The NMR spectrum of compound **1** exhibited two *N*-methyl signals [δ_H_ 2.27, 2.62 (s) H-2, H-2′] and four *O*-methyl signals [δ_H_ 3.15, 3.37, 3.73, 3.89 (s) CH_3_O-8, CH_3_O-7′, CH_3_O-7, CH_3_O-15] (Supplementary Information Figure S2). Furthermore, compound **1** is an isomer of well-known dauricine’s analogues [18,19,20]. The structure of dauricine was established as a head-to-tail BBIQA by spectroscopic data [21]. However, the HMBC spectrum of **1** showed the expected C−H correlations via two and three bonds within the two tetrahydroisoquinoline units, but gave no additional information about the connectivity of the two moieties (head-to-tail, tail-to-tail, or head to head; no correlations via four bonds were observed; SI Figure S9). In addition, H-11/11′ correlated with the aromatic carbons C-10/10′, C13/13′ and C17/17′ (Supplementary Information Figure S9). Analysis of the ROESY spectrum suggested the methoxy position to aromatic rings [correlations between H-6, CH_3_O-7, and H-6′, CH_3_O-7′, and H-16, CH_3_O-15, respectively; Supplementary Information Figure S10]. Collectively, these data indicate the structure of the new compound Lunagrad A (**1**) as shown in Figure 1.

**Figure 3:**
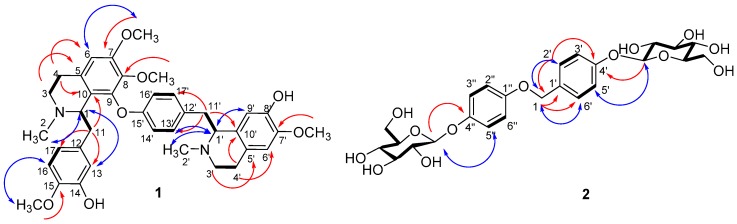
Key COSY (thick lines), HMBC (H➞C) and ROESY (↔) correlations of **1 **and** 2**.

Lunagrad B (**2**) was obtained as a white amorphous powder. The molecular formula, C_25_H_32_O_13_, which requires ten degrees of unsaturation, was determined on the basis of the [M + H]^+^ ion peak at *m*/*z* = 541.1917 (calcd. 541.1921) in the HR-MS (ESI) . The acid hydrolysis of **2** gave D-glucose, which was identified after derivatization by GC-MS. The β stereochemistry was determined by the coupling constants of the anomeric hydrogen (7.2 Hz). The ^1^H and ^13^C-NMR (CD_3_OD) spectra of **2** (Supplementary Information Figures S11 and S13), which were assigned by two-dimensional (2D) gradient NMR experiments (HSQC, HMBC, COSY) (Supplementary Information Figures S14–S17), showed benzyloxy protons [δ 4.54 (2H, s) H-1], and two A_2_B_2_ systems specific to a *para*-substituted aromatic pattern [δ 7.02, 7.27 (2H each, both d, *J* = 8.4 Hz) H-2′/6′, H-3′/5′; δ 6.69, 6.96 (2H each, both d, *J* = 8.8 Hz) H-2″/6″, H-3″/5″] (Supplementary Information Figure S14). In the HMBC experiment of **2**, H-1 correlated with the aromatic C-1′ and C-2′/6′ (δ_C_ 136.6, 129.5). The chemically equivalent H-2′/6′ aromatic protons correlated with C-1 (δ_C_ 64.9), C-1′ (δ_C_ 136.6) and the oxygenated C-4′ (δ_C_ 158.5). In addition, the chemically equivalent H-2″/6″ aromatic protons correlated with the oxygenated C-1″/4″ (δ_C_ 152.5, 153.9) (Supplementary Information Figure S16). The HSQC spectrum clearly showed two glucopyranosyl pattern and two correlation between the anomeric proton (δ 4.73) [(2H, d, *J* = 7.2 Hz), H-4′-O-Glu-1 and H-4″-O-Glu-1] and the anomeric carbons (δ_C_ 102.4, 103.7) (Supplementary Information Figure S15). On the basis of these findings, the structure of the new compound Lunagrad B (**2**) was formulated as shown in Figure 3.

## 3. Experimental Section

### 3.1. General

GC-MS and EIMS, Trace GS Ultra equipped with a capillary TR-5MS SQC column (0.25 μm, 15 m × 0.25 mm i.d.) and DSQII Thermo Scientific mass spectrometer. High resolution mass spectra were measured at Keecloud Mass Spectrometry Service Company on a Thermo Scientific LTQ Orbitrap XL system. Optical rotations were measured on a JASCO-1020 polarimeter (JASCO, Tokyo, Japan). Infrared spectra were collected on a Bruker model TENSOR27 spectrophotometer (Bruker Optics, Ettlingen, Germany). All of the NMR spectra were obtained using a Bruker AVANCE III 500 spectrometer (Bruker BioSpin, Rheinstetten, Germany). Sephadex LH-20 (25–100 μm, Pharmacia Fine Chemical Co., Ltd., Uppsala, Sweden), D101/D201 macroporous adsorption resin (Dalian Elite Analytical Instrument Co. Ltd., Dalian, China), MCI GEL CHP20P (Mitsubishi Chemical Co., Tokyo, Japan), C18 reversed-phase silica gel (230–400 mesh, Sigma-Aldrich, St. Louis, MO, USA), Silica gel (200–300 mesh), and Silica gel H (Qingdao Oceanic Chemical Co., Ltd., Qingdao, China) were used for column chromatography. Thin-layer chromatography (TLC) was performed on glass-backed plates coated with 0.25 mm layers of Silica gel H (Qingdao Oceanic Chemical Co., Qingdao, China). Fractions were monitored by TLC and spots were visualized by heating silica gel plates sprayed with 5% H_2_SO_4_ in EtOH/H_2_O (1/1). All of the solvents and chemicals that were used were of analytical reagent grade (Sinopharm Chemical Reagent Co., Ltd., Beijing, China), and water was doubly distilled before use.

### 3.2. Plant Material

*Cymbidium* Lunagrad Eternal Green was purchased from Fujian Flower Market, Fujian Province, People’s Republic of China, in September 2012 and identified by Prof. Shi-Pin Chen, Forestry College, Fujian Agriculture and Forestry University. A voucher specimen (No. 070612) was deposited in the Forestry College, Fujian Agriculture and Forestry University.

### 3.3. Extraction and Isolation

The flower organs of the plant (air-dried, 36 g) were extracted with MeOH (2 L, 3 d) at room temperature for three times and the combined organic phase was concentrated in *vacuum via a rotavap*. The residue was subjected to D101 macroporous adsorption resin and then filtered to give 9 g of crude extract. The extract was subjected to D201 macroporous adsorption resin with a H_2_O/MeOH elution gradient (from 100% H_2_O to 100% MeOH) to yield 10 fractions (Fractions 1–10). 

Fraction 2 (608 mg) was subjected to silica gel column chromatography (CHCl_3_/MeOH, 20:1) to give **10** (18.7 mg, *R*_f_ = 0.20, CHCl_3_/MeOH = 10:1).

Fraction 3 (1.30 g) was subjected to Sephadex LH-20 column chromatography eluted with MeOH to afford four fractions 3-1–3-4. Fraction 3-1 was further purified by RPC column chromatography (MeOH/H_2_O, 1:1) to afford **1** (60.7 mg). Fraction 3-3 was purified by MCI column chromatography (MeOH/H_2_O, 3:5 to 1:0) to afford **6** (20.3 mg). Fraction 3-4 was obtained as white powder (**2**, 26.4 mg). A solution of **2** in 2M hdrochloric acid (0.5 mL) was heated to reflux for 3 h. The solution was extracted with *t*-butyl alcohol. The dried residue of the aqueous layer was derivatized by pyridine and 1-(trimethylsilyl)-imidazole at 60 °C for 1 h. The mixture was analyzed by GC-MS (Trace GS Ultra) with a capillary TR-5MS SQC column (0.25 μm, 15 cm × 0.25 mm) under the following conditions: 1 min at 40 °C, increase of temperature with a thermal ramp of 10 °C/min until 250 °C (helium flow rate 1 mL/min, injector temperature 250 °C, transfer temperature 285 °C). Impact electronic detection was performed with a DSQII Thermo Scientific mass spectrometer. Identification of d-glucose was carried out by a comparison of its retention time and spectroscopic data with those of an authentic sample of d-glucose (Purchased from Aldrich), *t*_R_ 14.8 min.

Fraction 4 (1.50 g) was subjected to D201 macroporous adsorption resin with a H_2_O/MeOH elution gradient (H_2_O/MeOH, 9:1 to 1:4) to yield 6 fractions 4-1–4-6. Fraction 4-3 was obtained as white powder (**5**, 39.2 mg). Fraction 4-4 was subjected to silica gel column chromatography (petroleum ether/acetone, 10:1 to 1:1) to yield **4** (23.2 mg, *R*_f_ = 0.26, CHCl_3_/MeOH = 10:1) and **9** (30.1 mg). Fraction 4-5 was purified by MCI column chromatography eluting with MeOH to afford **8** (12.6 mg).

Fraction 7 (1.20 g) was subjected to silica gel column chromatography (CHCl_3_/MeOH, 30:1 to 3:1) to yield 6 fractions 7-1–7-6. Fraction 7-2 was obtained as a white powder (**3**, 25.0 mg). Fraction 7-3 was purified by MCI column chromatography (H_2_O/MeOH, 2:3), to afford **7** (39.4 mg).

Fraction 8 (910 mg) was subjected to silica gel column chromatography (petroleum ether/acetone, 20:1 to 2:1) to yield 5 fractions 8-1–8-5. Fraction 8-5 was obtained as yellow powder (**11**, 19.6 mg).

### 3.4. Spectral Data

Lunagrad A (**1**). Slightly yellow, amorphous powder. [α]${}_{D}^{25}$ +11.4 (*c* 0.1, MeOH). IR (KBr) *ν*_max_ 3420, 1617, 1509, 1463, 1270, 1222, 1169, 1125, 1076 cm^−1^. HR-MS (ESI) *m*/*z* calculated for C_38_H_45_N_2_O_7_ [M + H]^+^ 641.3227, found 641.3230. ^1^H-NMR (500 MHz, CD_3_OD) and ^13^C-NMR (125 MHz, CD_3_OD) data (see Table 1).

Lunagrad B (**2**). White, amorphous powder. [α]${}_{D}^{25}$ −37.8 (*c* 0.1, MeOH). IR (KBr) *ν*_max_ 3230, 1610, 1450, 1221, 1188, 1095 cm^−1^. HR-MS (ESI) *m*/*z* calculated for C_25_H_33_O_13_ [M + H]^+^ 541.1921, found 541.1917. ^1^H-NMR (500 MHz, CD_3_OD) and ^13^C-NMR (125 MHz, CD_3_OD) data (see Table 2).

## 4. Conclusions

The first chemical investigation of the flowers of *Cymbidium* Lunagrad Eternal Green resulted in the isolation of a new alkaloid, named Lunagrad A (**1**), and a new aromatic glucoside, named Lunagrad B (**2**), together with nine known compounds, **3**–**11**. These specific compounds provided further confirmation of the typical profile of secondary metabolites that are found in the orchid family, and might be useful for further biological studies.

## Figures and Tables

**Table 1:** ^1^H-NMR (500 MHz) and ^13^C-NMR (125 MHz) data of compound **1** in CD_3_OD (δ, ppm; *J*, Hz) (Supplementary Information Figures S2 and S3).

No.		^1^H-NMR	^13^C-NMR	No.		^1^H-NMR	^13^C-NMR
1	CH	3.82, d, 10.0	63.0	1′	CH	4.00, dd, 11.0, 6.0	64.5
2	CH_3_	2.27, s	43.1	2′	CH_3_	2.62, s	42.5
3	CH_2_	3.49, overlap 2.91, overlap	45.3	3′	CH_2_	3.47, m 2.78, overlap	45.7
4	CH_2_	2.92, overlap 2.53, dd, 14.5, 4.5	23.7	4′	CH_2_	3.01, m 2.86, overlap	26.0
5	C	-	124.0	5′	C	-	129.1
6	CH	6.42, s	107.3	6′	CH	6.66, s	113.7
7	C	-	153.0	7′	C	-	150.3
7-OMe	CH_3_	3.73, s	56.4	7′-OMe	CH_3_	3.37, s	56.1
8	C	-	139.3	8′	C	-	145.2
8-OMe	CH_3_	3.15, s	60.7			-	
9	C	-	149.4	9′	CH	6.01, s	121.4
10	C	-	129.3	10′	C	-	129.0
11	CH_2_	2.76, dd, 14.5, 10.0 2.46, d, 14.5	42.6	11′	CH_2_	3.27, dd, 12.5, 6.0 2.87, overlap	37.5
12	C	-	135.8	12′	C	-	136.7
13	CH	6.56, d, 1.6	116.7	13′	CH	7.43, d, 8.2	131.8
14	C	-	151.0	14′	CH	7.05, d, 8.2	122.9
15	C	-	148.7	15′	C	-	155.1
15-OMe	CH_3_	3.89, s	56.8	-	-	-	-
16	CH	6.92, d, 8.2	113.2	16′	CH	6.76, d, 8.2	122.8
17	CH	6.86, d, 8.2	124.4	17′	CH	6.38, d, 8.2	133.9

**Table 2:** ^1^H-NMR (500 MHz) and ^13^C-NMR (125 MHz) data of compound **2** in CD_3_OD (δ, ppm; *J*, Hz) (Supplementary Information Figures S11 and S12).

No.		^1^H-NMR	^13^C-NMR	No.		^1^H-NMR	^13^C-NMR
1′	C	-	136.6	1″	C	-	153.9
2′, 6′	CH	7.27, d, 8.4	129.5	2″, 6″	CH	6.69, d, 8.8	116.6
3′, 5′	CH	7.07, d, 8.4	117.7	3″, 5″	CH	6.96, d, 8.8	119.4
4′	C	-	158.5	4″	C	-	152.5
4′-O-Glu-1	CH	4.89, d, 7.2	102.4	4″-O-Glu-1	CH	4.73, d, 7.2	103.7
4′-O-Glu-2	CH	3.42, overlap	75.1	4″-O-Glu-2	CH	3.42, overlap	75.0
4′-O-Glu-3	CH	3.41, overlap	78.2	4″-O-Glu-3	CH	3.41, overlap	78.0
4′-O-Glu-4	CH	3.34, overlap	71.5	4″-O-Glu-4	CH	3.34, overlap	71.4
4′-O-Glu-5	CH	3.36, overlap	78.1	4″-O-Glu-5	CH	3.36, overlap	78.0
4′-O-Glu-6	CH_2_	3.88, br.d, 12.0 3.69, br.d, 12.0	62.6	4″-O-Glu-6	CH_2_	3.88, br.d, 12.0 3.69, br.d, 12.0	62.5
1	CH_2_	4.54, s	64.9	-	-	-	-

## Supplementary Materials

Copies of ^1^H, ^13^C, DEPT, COSY, HSQC, HMBC and ROESY spectra of new compounds. This material is available free of charge online.
