# Supplementary material for: New Alkaloid and Aromatic Glucoside from the Flowers of Cymbidium Lunagrad Eternal Green
Source: Molecules. 2018 Jan 3;23(1):99. doi: 10.3390/molecules23010099 (PMC6017434; doi:10.3390/molecules23010099)

Supporting Information

New alkaloid and aromatic glucoside from the flowers of *Cymbidium* Lunagrad Etrnal Green

Li-Yan Song, Fang Huang, Yan Wang, Zu-Jian Wu and Ming-An Ouyang*
State Key Laboratory of Ecological Pest Control for Fujian and Taiwan Crops, Institute of Plant Virology, Fujian Agriculture and Forestry University, Fuzhou, Fujian, China
minganouyang@163.com

Table of Content

^{1}H NMR spectrum (500 MHz) of Lunagrad A (1) in CD_3OD.	S-2
^{13}C NMR spectrum (125 MHz) of Lunagrad A (1) in CD_3OD.	S-3
DEPT of Lunagrad A (1) in CD_3OD.	S-4
^{1}H-^{1}H COSY of Lunagrad A (1) in CD_3OD.	S-6
HSQC of Lunagrad A (1) in CD_3OD.	S-7
HMBC of Lunagrad (1) A in CD_3OD.	S-8
ROESY of Lunagrad (1) A in CD_3OD.	S-10
^{1}H NMR spectrum (500 MHz) of Lunagrad B (2) in CD_3OD.	S-11
^{13}C NMR spectrum (125 MHz) of Lunagrad B (2) in CD_3OD.	S-12
DEPT of Lunagrad B (2) in CD_3OD.	S-13
^{1}H-^{1}H COSY of Lunagrad B (2) in CD_3OD.	S-14
HSQC of Lunagrad B (2) in CD_3OD.	S-15
HMBC of Lunagrad B (2) in CD_3OD.	S-16
ROESY of Lunagrad B (2) in CD_3OD.	S-17

^{1}H NMR spectrum (500 MHz) of Lunagrad A (1) in CD_3OD.

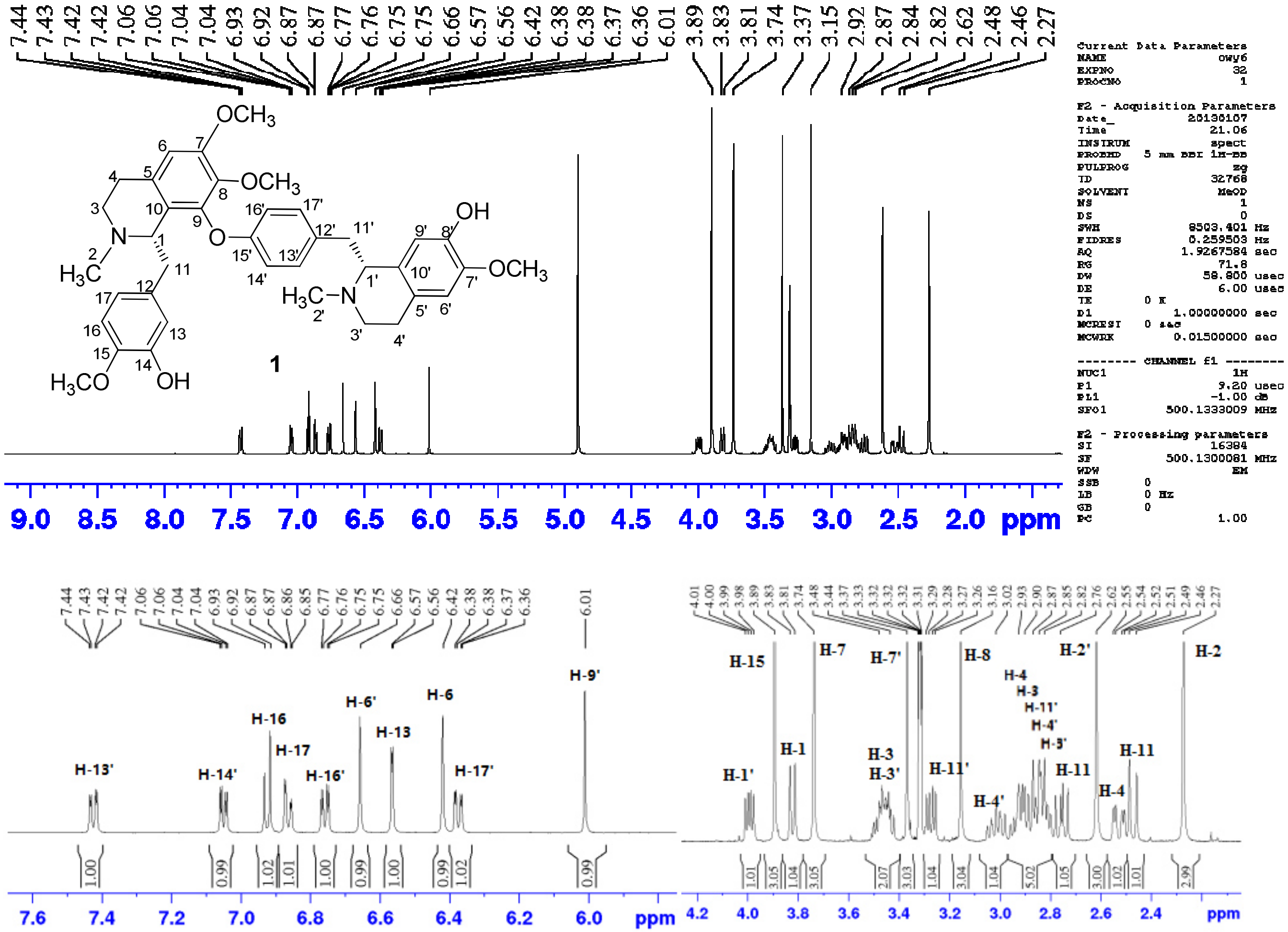

^{13}C NMR spectrum (125 MHz) of Lunagrad A (1) in CD_3OD.

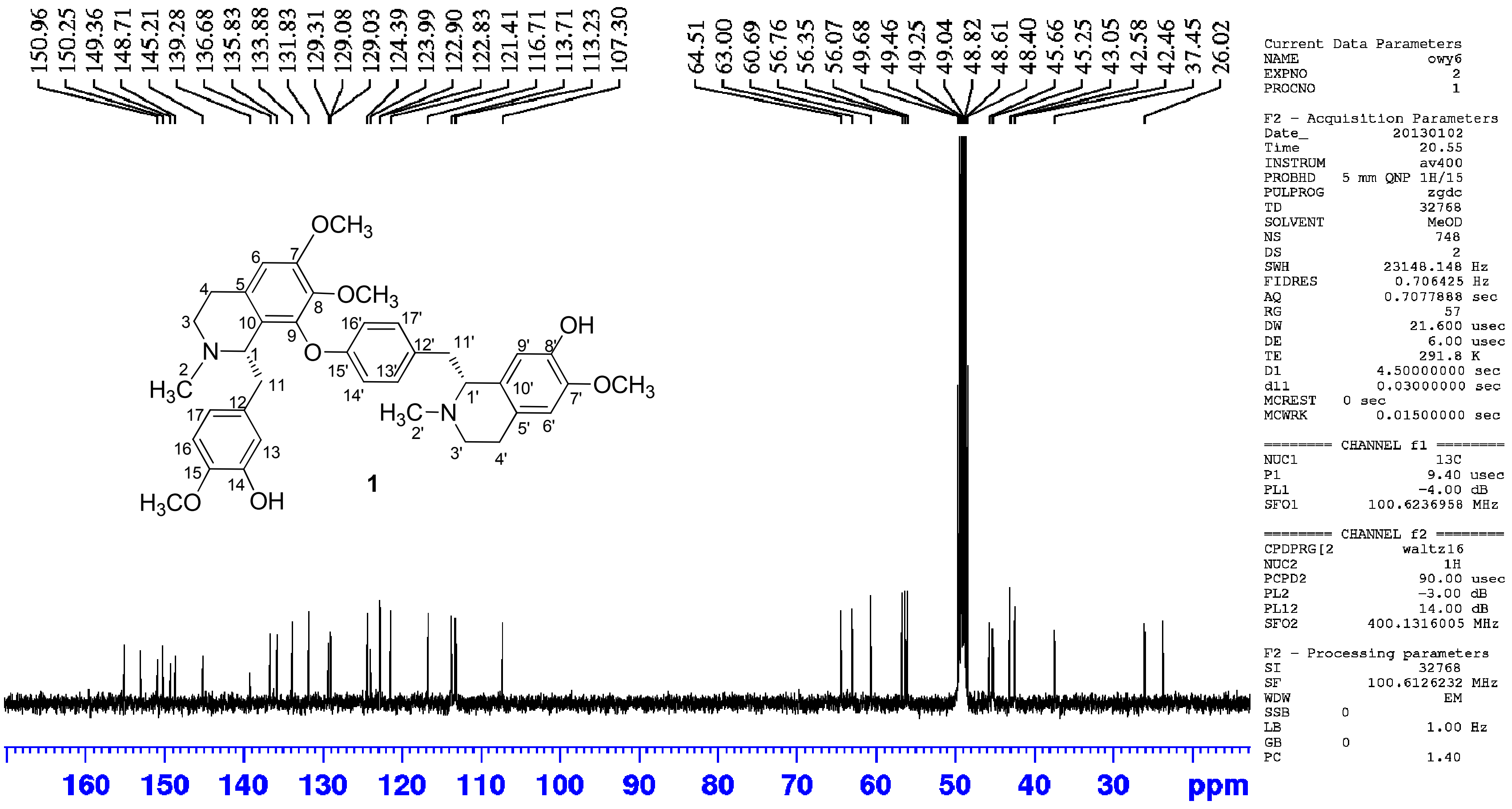

DEPT of Lunagrad A (1) in CD_3OD.

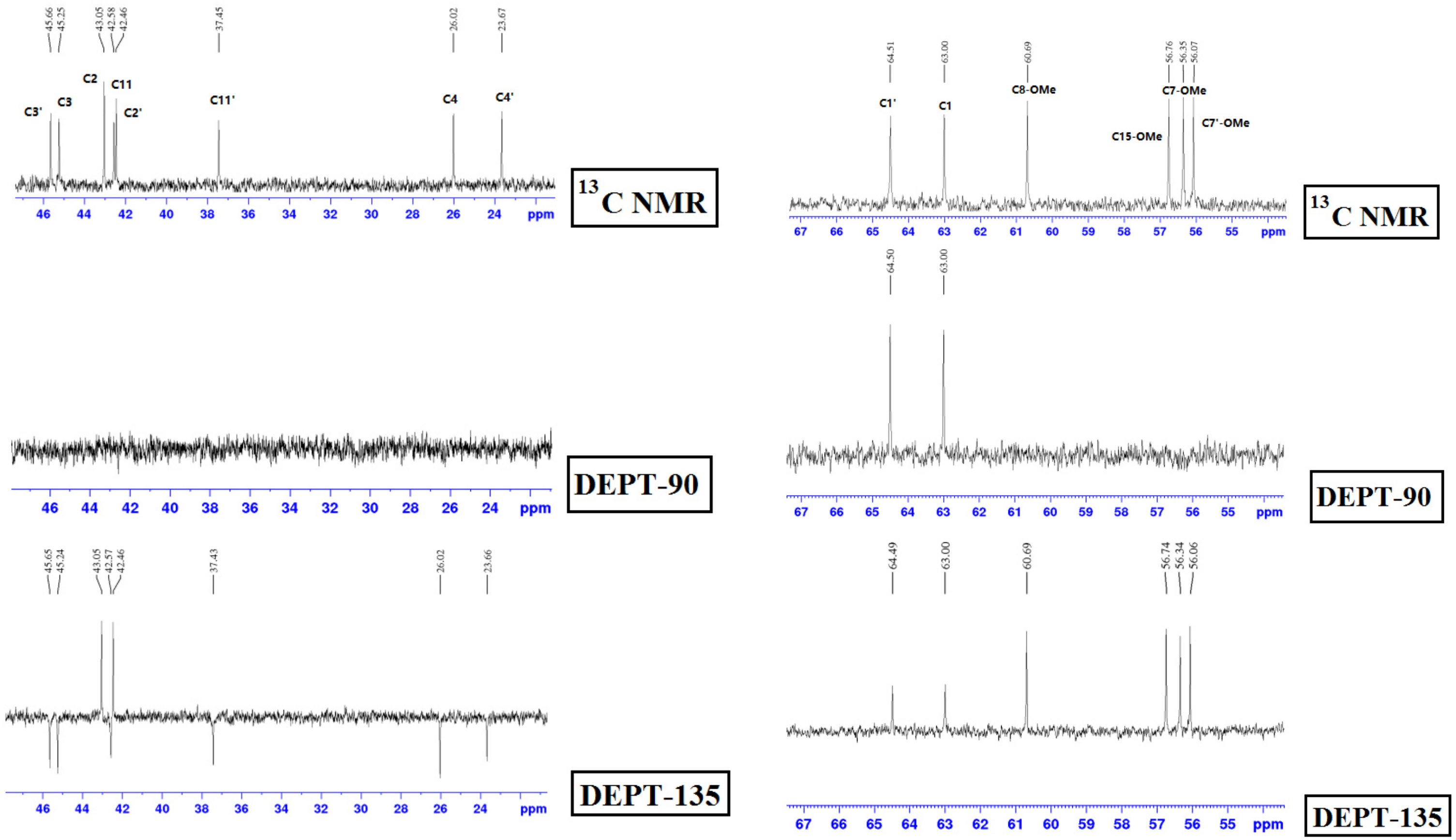

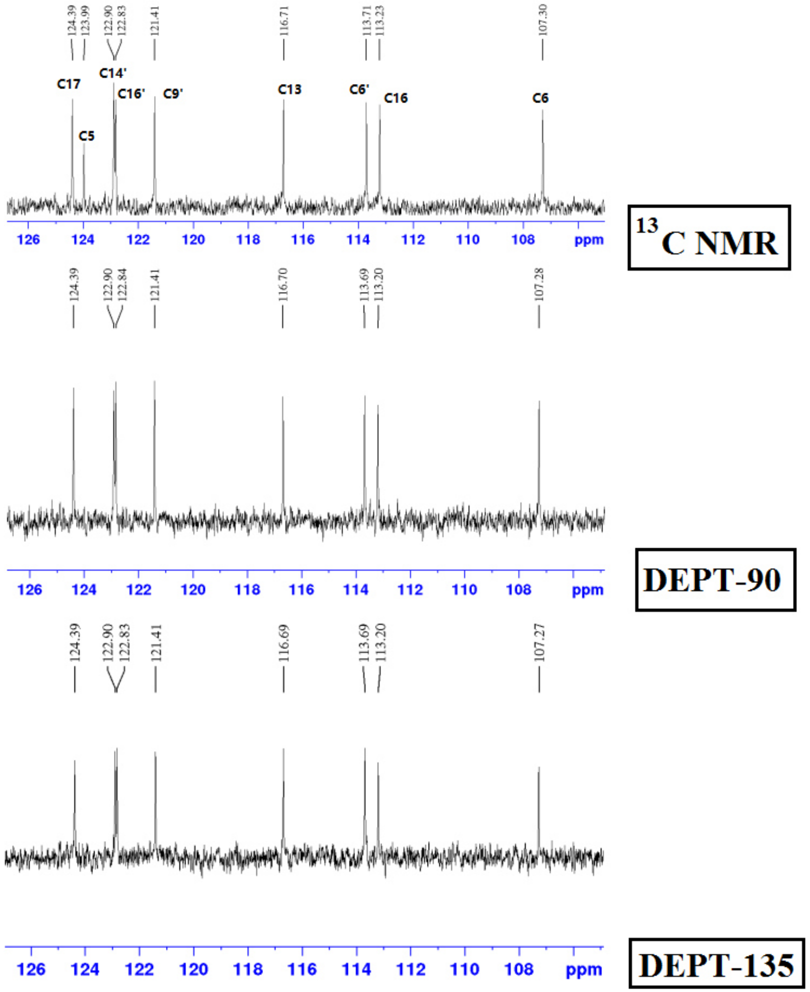
124.39
123.99
122.90
122.83
121.41
116.71
113.71
113.23
107.30
C17
C5
C14'
C16'
C9'
C13
C6'
C16
C6
126 124 122 120 118 116 114 112 110 108 ppm
13C NMR
124.39
122.90
122.84
121.41
116.70
113.69
113.20
107.28
126 124 122 120 118 116 114 112 110 108 ppm
DEPT-90
124.39
122.90
122.83
121.41
116.69
113.69
113.20
107.27
126 124 122 120 118 116 114 112 110 108 ppm
DEPT-135

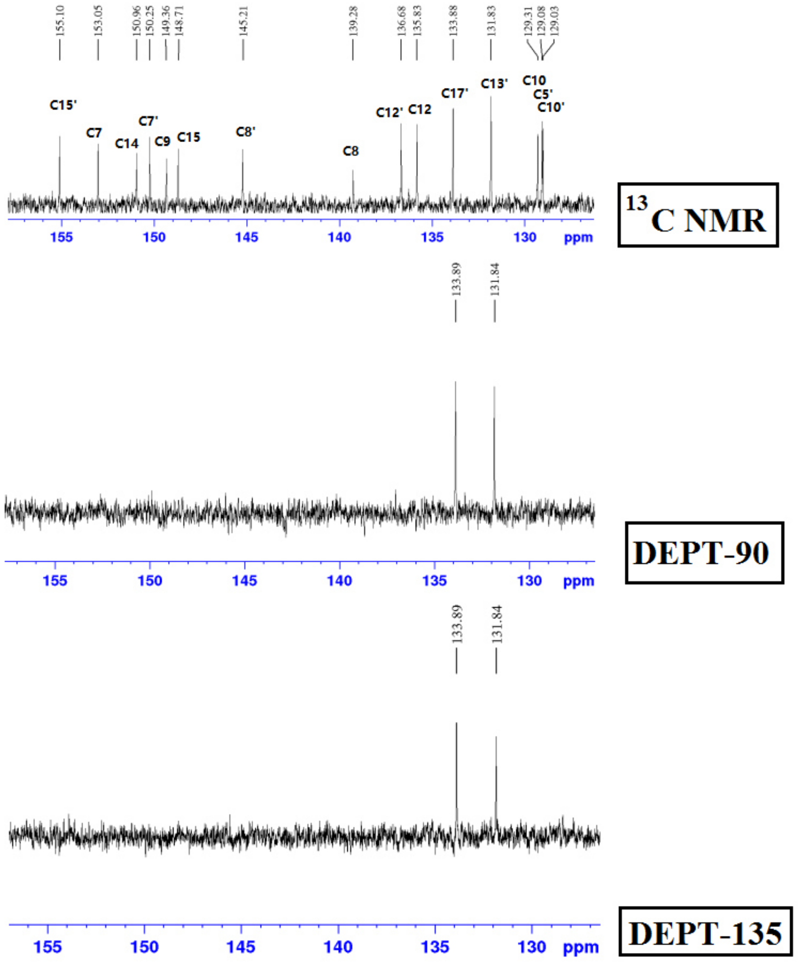
155.10
153.05
150.96
150.25
149.36
148.71
145.21
139.28
136.68
135.83
133.88
131.83
129.31
129.08
129.03
C15'
C7
C14
C7'
C9
C15
C8'
C8
C12'
C12
C17'
C13'
C10
C5'
C10'
155 150 145 140 135 130 ppm
13C NMR
133.89
131.84
155 150 145 140 135 130 ppm
DEPT-90
133.89
131.84
155 150 145 140 135 130 ppm
DEPT-135

^{1}H-^{1}H COSY of Lunagrad A (1) in CD_3OD.

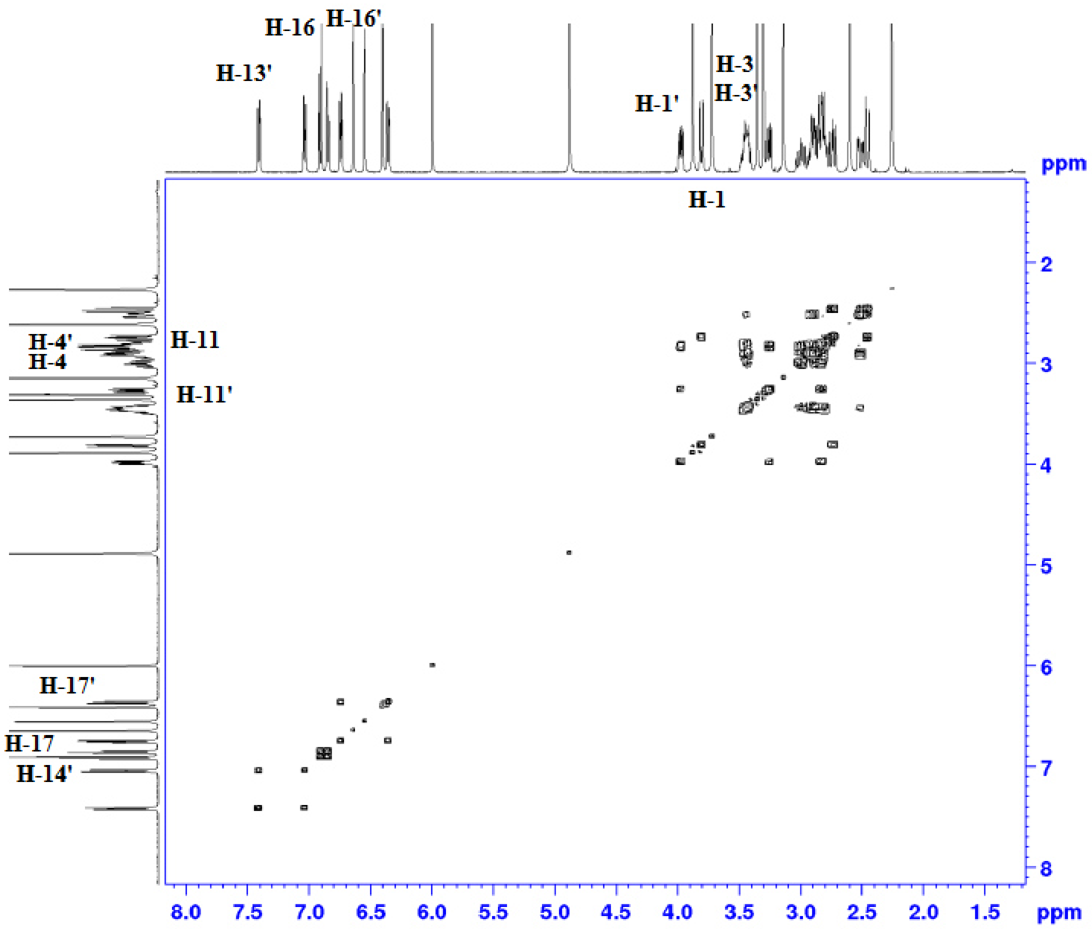


```
Current Data Parameters
NAME                owy6
EXPNO                 29
PROCNO                 1

F2 - Acquisition Parameters
Date_           20130107
Time               20.56
INSTRUM            spect
PROBHD   5 mm BBI 1H-BB
PULPROG        cosygpmfqf
TD                  1024
SOLVENT             MeOD
NS                     1
DS                    16
SWH             3501.401 Hz
FIDRES          3.419337 Hz
AQ             0.1462272 sec
RG                  6144
DW               142.800 usec
DE                  6.00 usec
TE         0 K
D0            0.00000300 sec
D1            1.00000000 sec
d13           0.00000400 sec
D16           0.00020000 sec
IN0           0.00028570 sec
MCREST     0 sec
MCWRK         1.00000000 sec

======== CHANNEL f1 ========
NUC1                  1H
P1                  9.20 usec
PL1                -1.00 dB
SFO1         500.1323506 MHz

====== GRADIENT CHANNEL =====
GPNAM[1]        sine.100
GPNAM[2]        sine.100
GPNAM[3]        sine.100
GPX1       0 %
GPX2       0 %
GPX3       0 %
GPY1       0 %
GPY2       0 %
GPY3       0 %
GPZ1               16.00 %
GPZ2               12.00 %
GPZ3               40.00 %
P16              1000.00 usec

F1 - Acquisition parameters
TD                   128
SFO1            500.0318 MHz
FIDRES         54.690235 Hz
SW                 7.000 ppm
FnMODE                QF

F2 - Processing parameters
SI                  1024
SF           500.1300140 MHz
WDW                 SINE
SSB        0
LB         0 Hz
GB         0
PC                  1.00

F1 - Processing parameters
SI                  1024
MC2                   QF
SF           500.0294140 MHz
WDW                 SINE
SSB        0
LB         0 Hz
GB         0
```

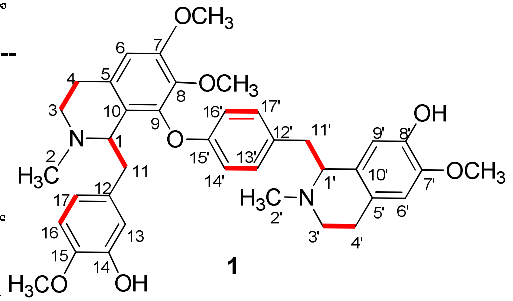

1

HSQC of Lunagrad A (1) in CD_3OD.

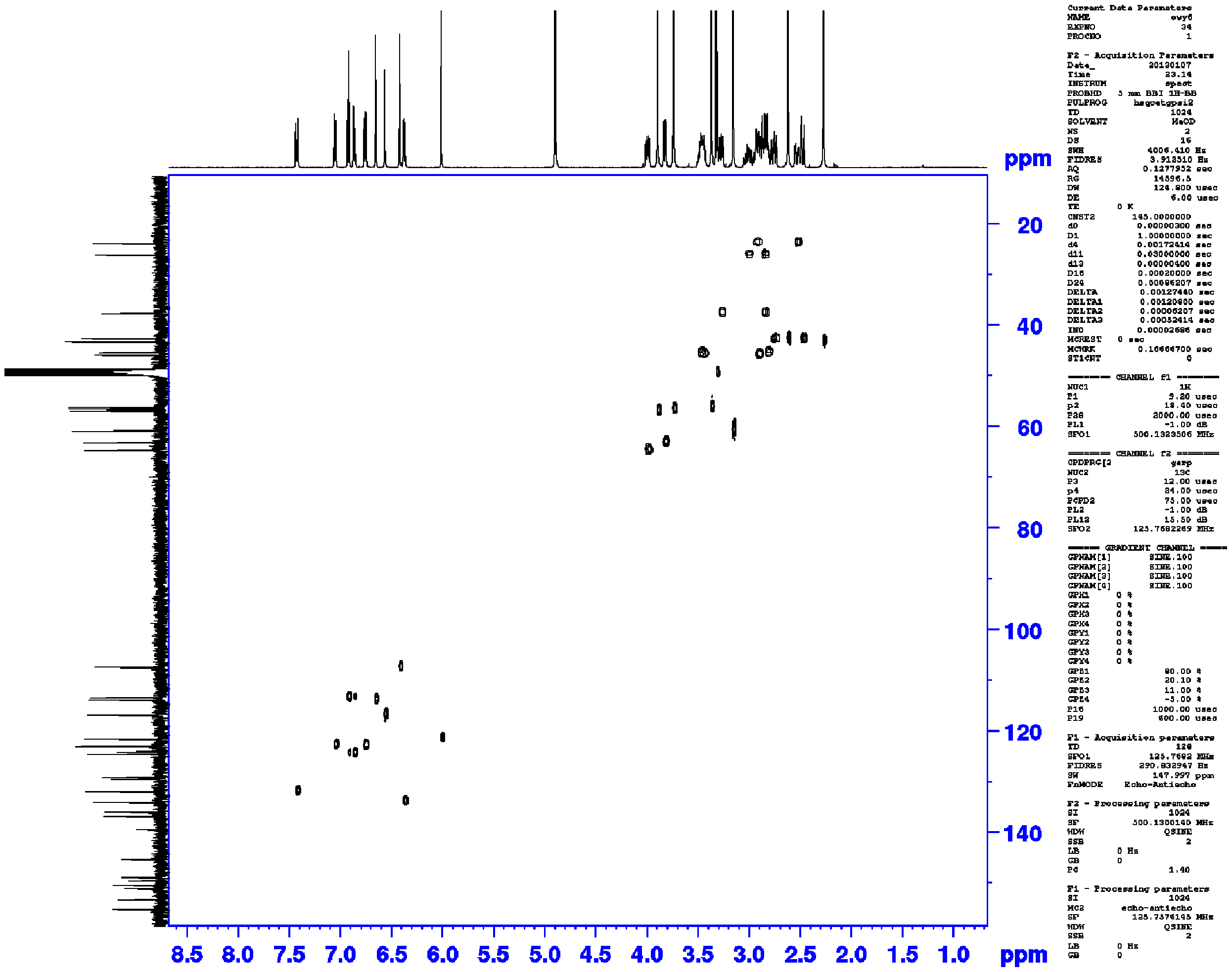

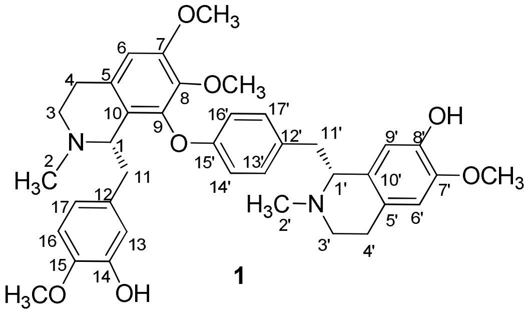

1

HMBC of Lunagrad A (1) in CD_3OD.

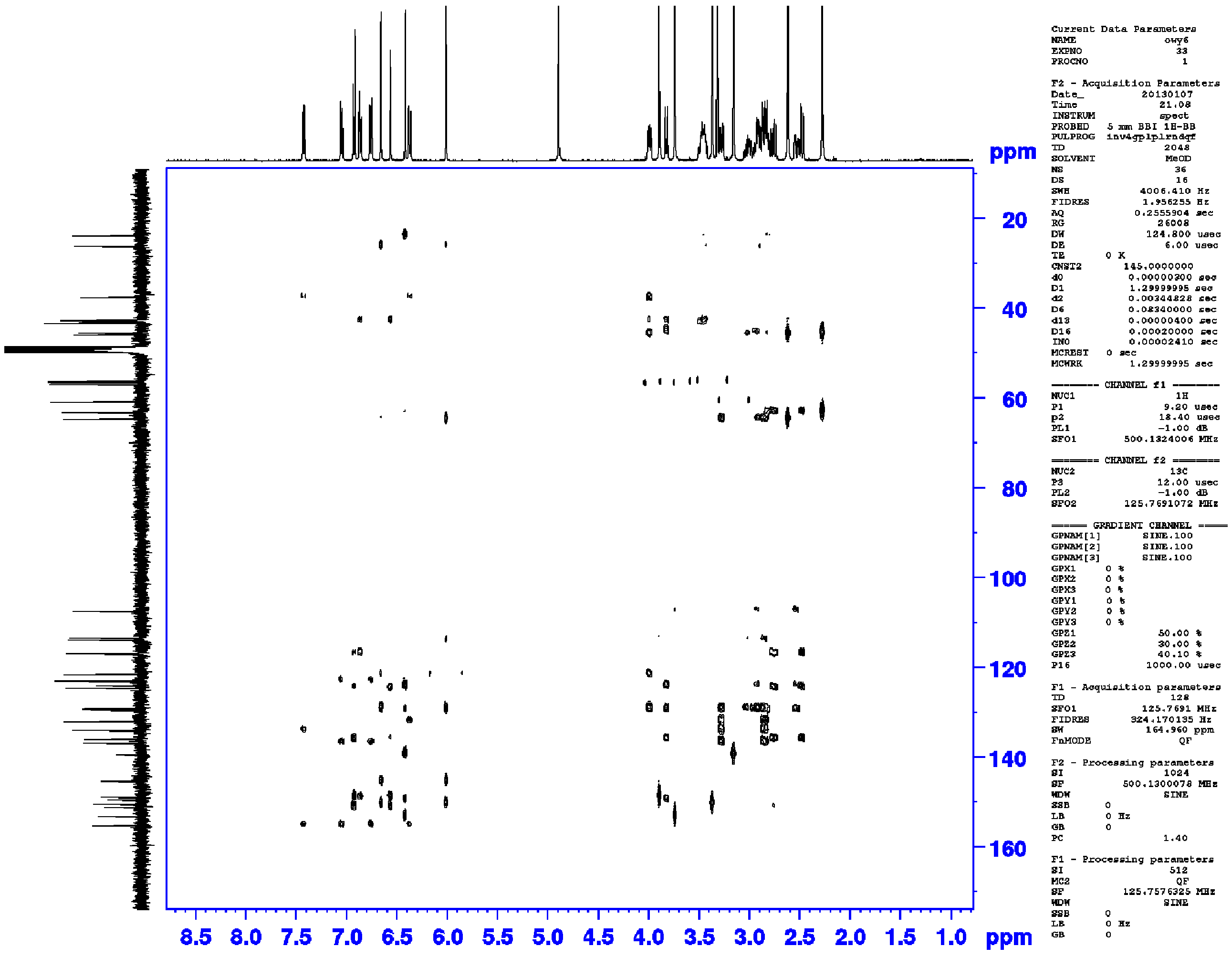

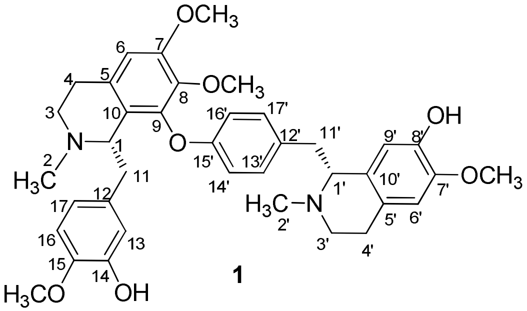

1

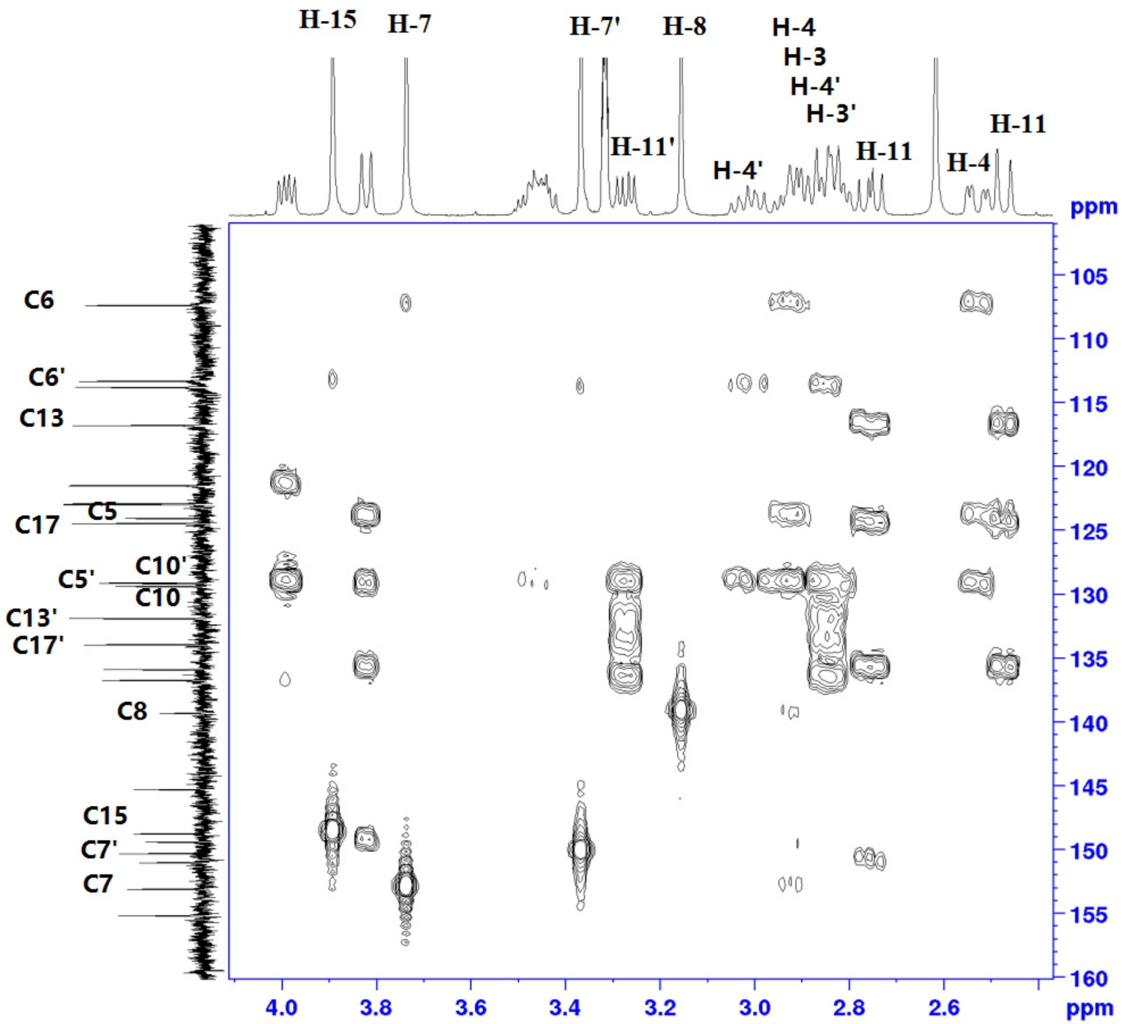
H-15
H-7
H-7'
H-11'
H-8
H-4'
H-4
H-3
H-4'
H-3'
H-11
H-4
H-11
C6
C6'
C13
C17
C5
C5'
C10'
C10
C13'
C17'
C8
C15
C7'
C7
ppm

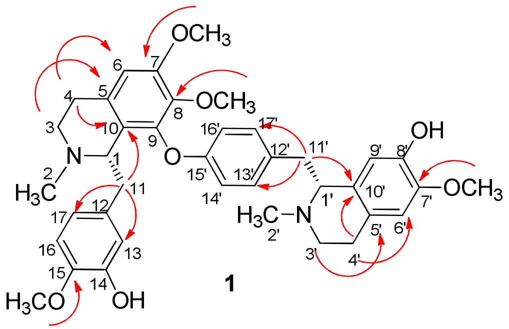

1

ROESY of Lunagrad A (1) in CD_3OD.

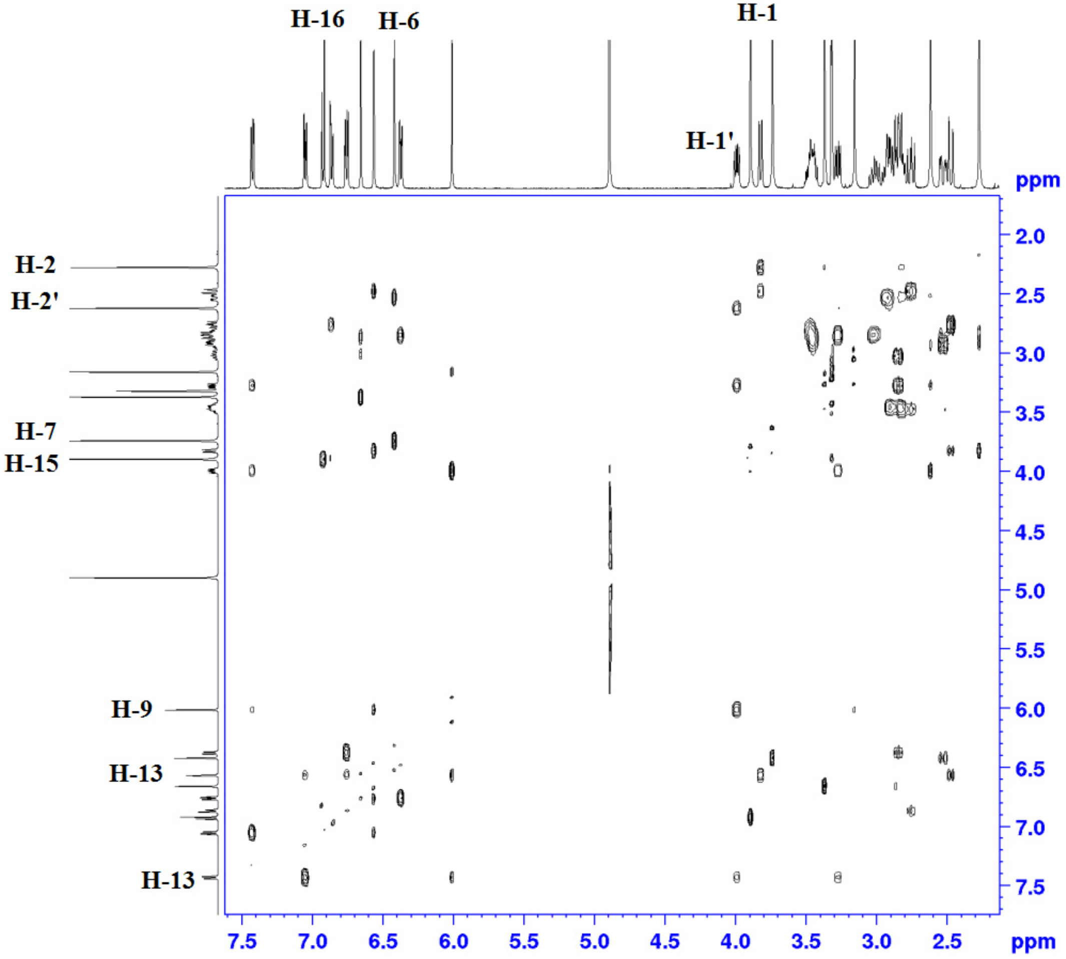


```
Current Data Parameters
NAME                owy6
EXPNO                 28
PROCNO                 1

F2 - Acquisition Parameters
Date_           20130107
Time               20.05
INSTRUM            spect
PROBHD   5 mm BBI 1H-BB
PULPROG        croesyph
TD                  1024
SOLVENT             MeOD
NS                     8
DS                     4
SWH         3501.401 Hz
FIDRES      3.419337 Hz
AQ         0.1462272 sec
RG                    28
DW          142.800 usec
DE             6.00 usec
TE          0 K
d0        0.00013110 sec
D1        1.00000000 sec
d12       0.00002000 sec
d13       0.00000400 sec
IN0       0.00028564 sec
MCREST    0 sec
MCWRK     0.50000000 sec
ST1CNT                 0

-------- CHANNEL f1 --------
NUC1                  1H
P1             9.20 usec
P15      1200000.00 usec
PL1            -1.00 dB
PL11           22.00 dB
SFO1     500.1323506 MHz

F1 - Acquisition parameters
TD                   160
SFO1         500.132 MHz
FIDRES     43.761761 Hz
SW             7.000 ppm
FnMODE       States-TPPI

F2 - Processing parameters
SI                  1024
SF       500.1300068 MHz
WDW                QSINE
SSB                    2
LB        0 Hz
GB        0
PC                  1.00

F1 - Processing parameters
SI                  1024
MC2          States-TPPI
SF       500.1296572 MHz
WDW                QSINE
SSB                    2
LB        0 Hz
GB        0
```

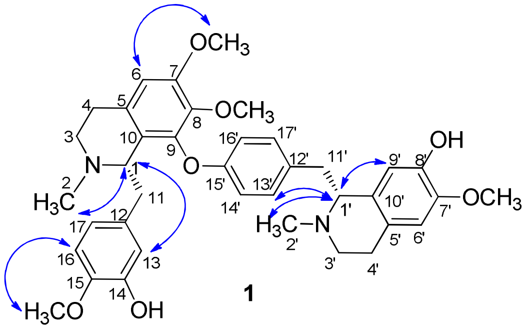

1

^{1}H NMR spectrum (500 MHz) of Lunagrad B (2) in CD_3OD.

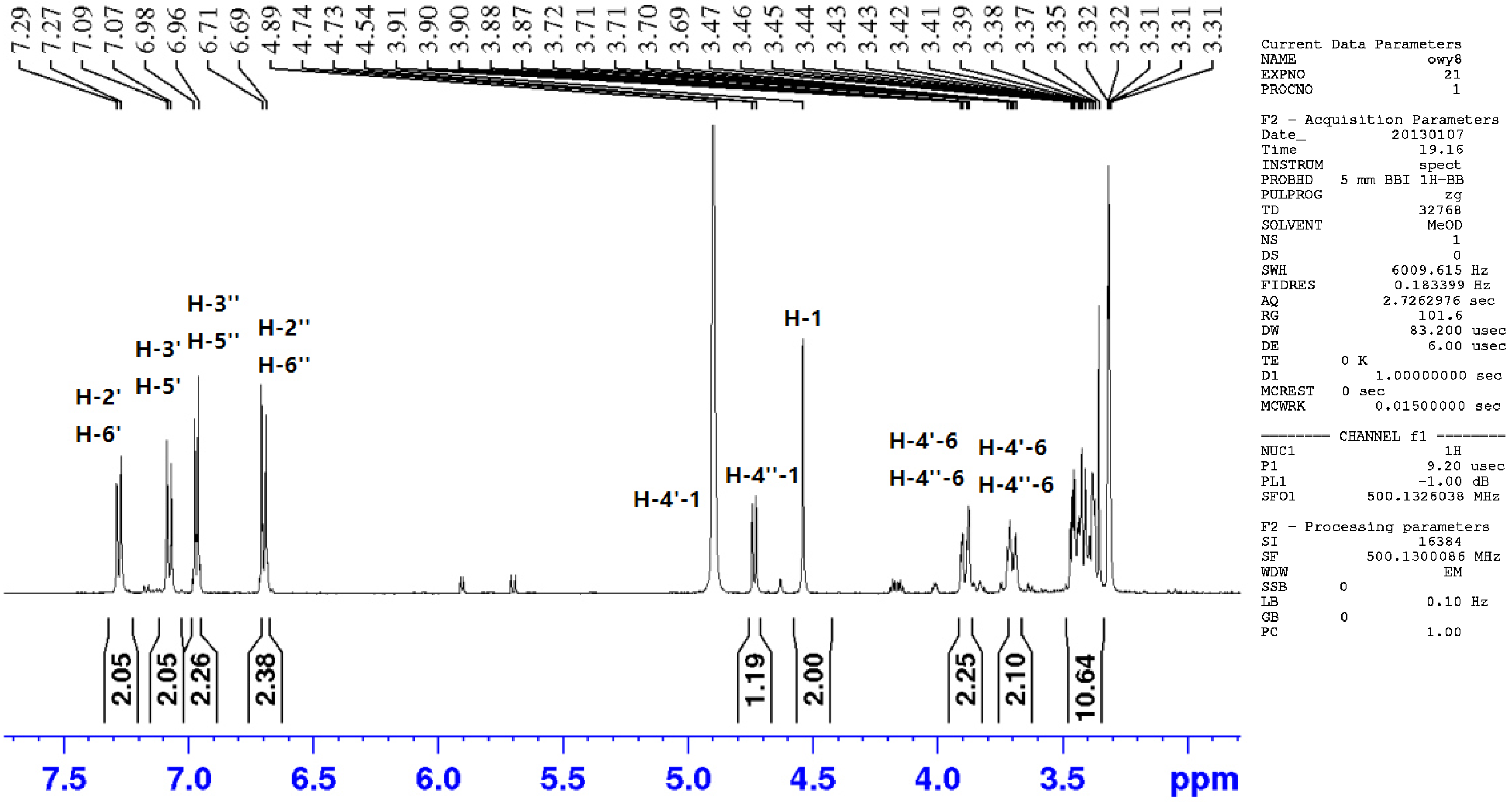

^{13}C NMR spectrum (125 MHz) of Lunagrad B (2) in CD_3OD.

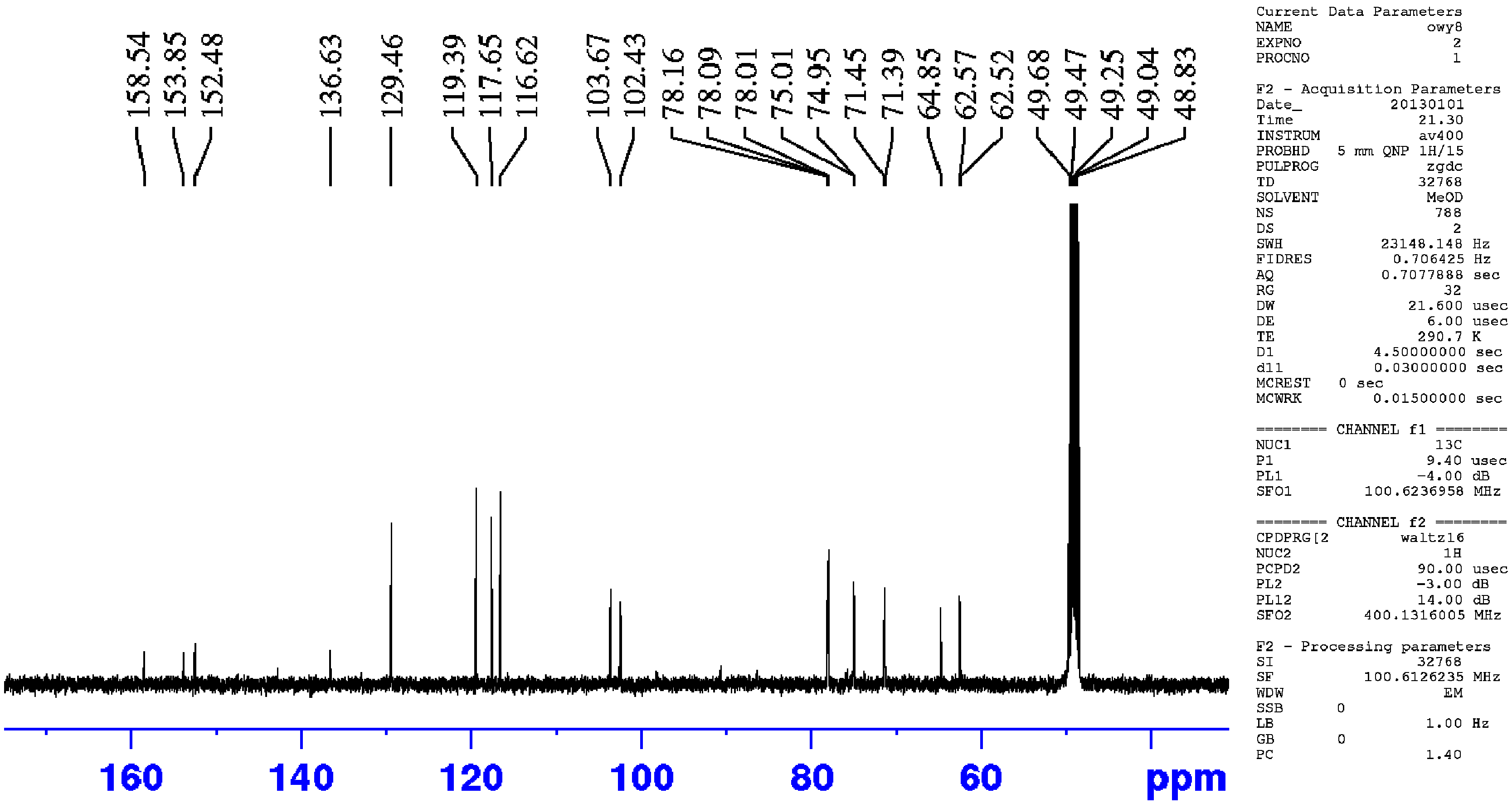

DEPT of Lunagrad B (2) in CD_3OD.

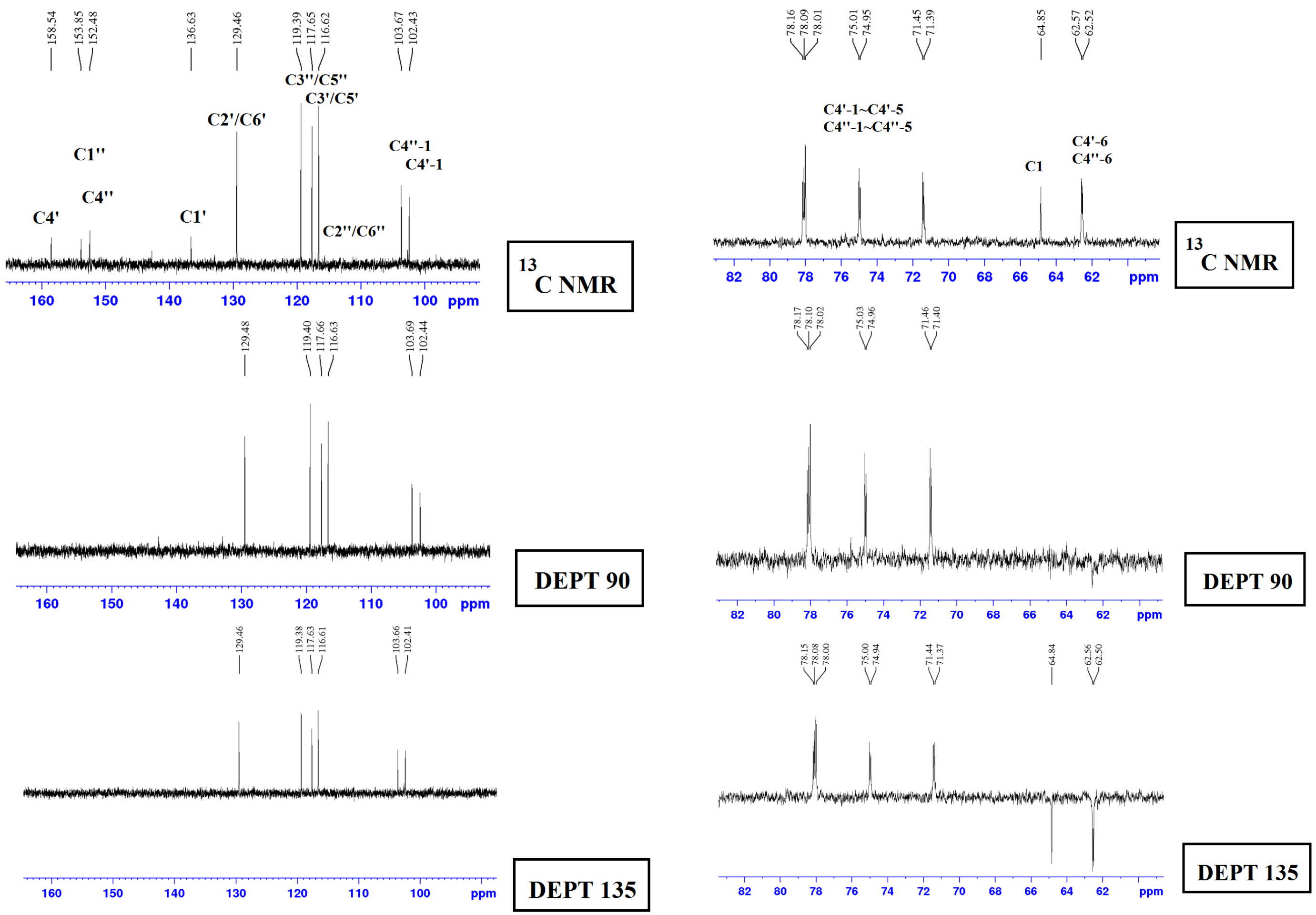

^{1}H-^{1}H COSY of Lunagrad B (2) in CD_3OD.

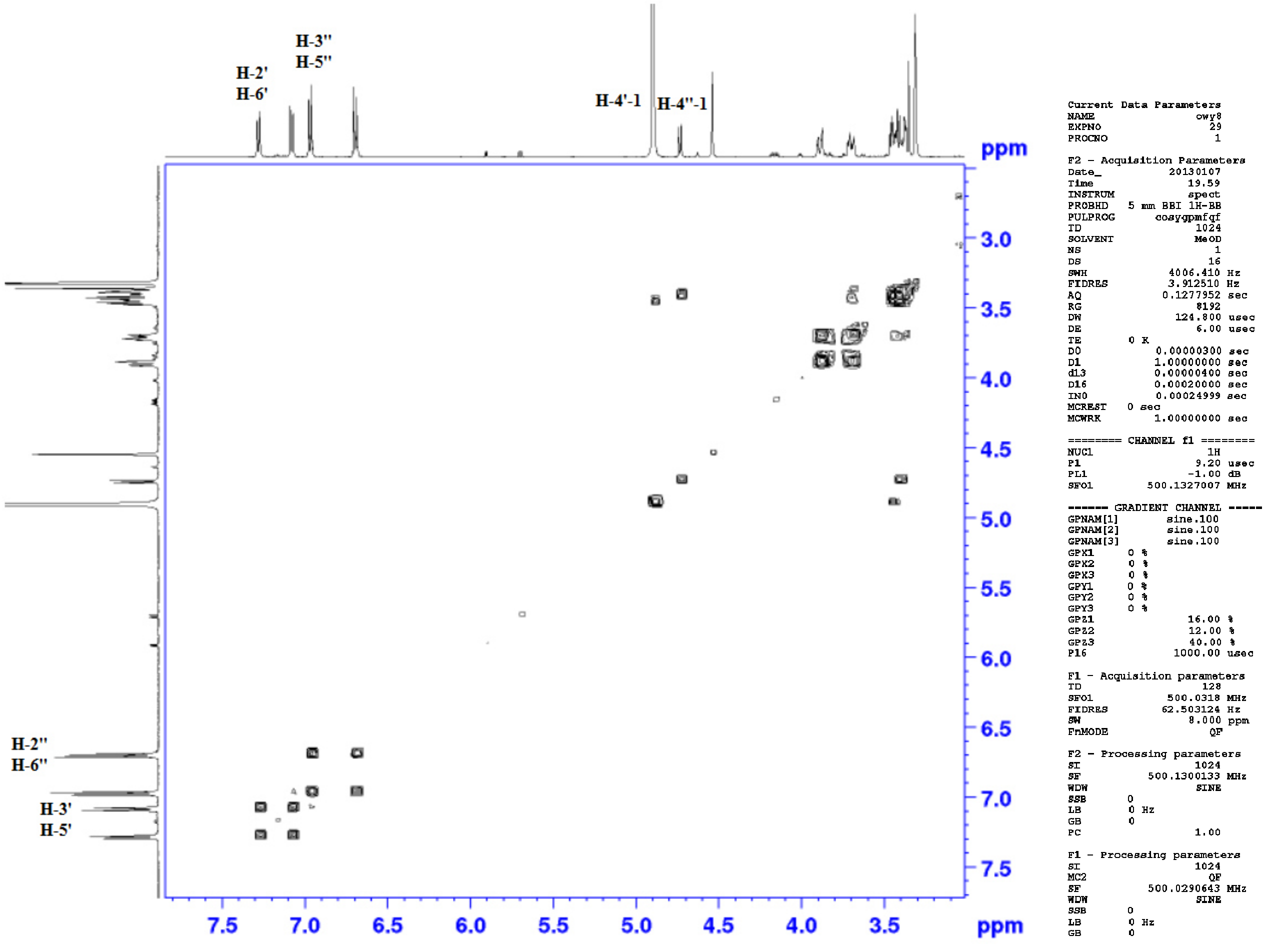

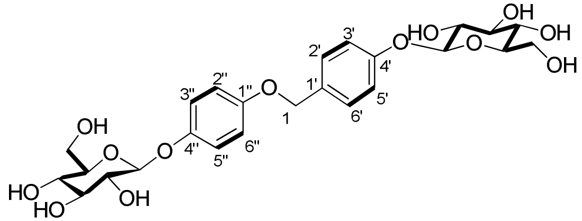

HSQC of Lunagrad B (2) in CD_3OD.

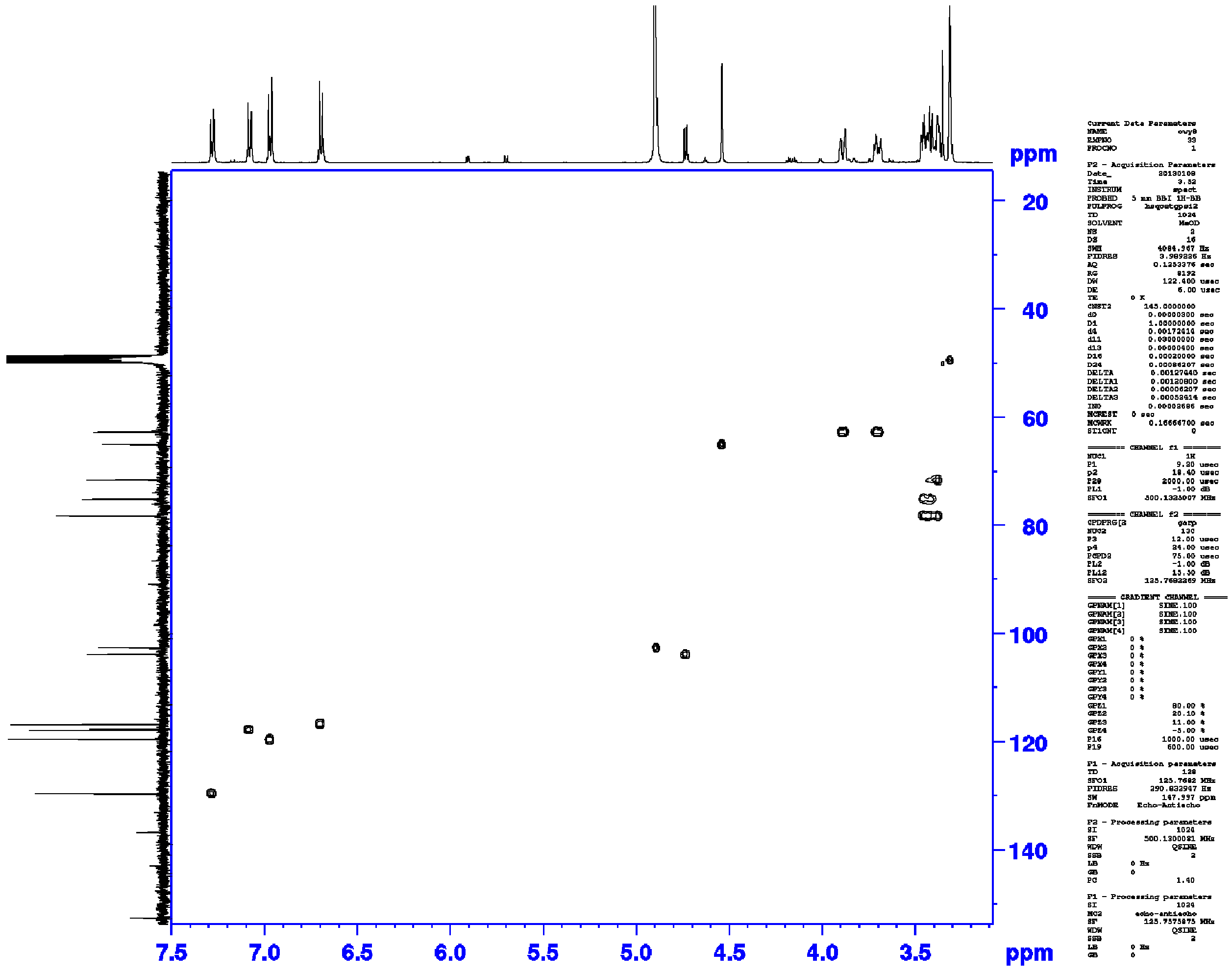

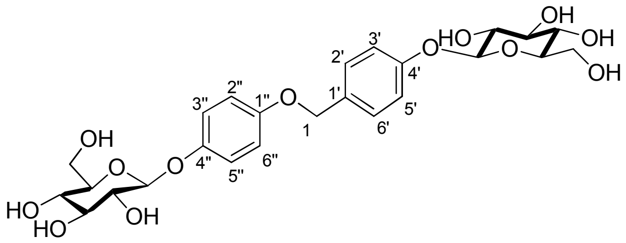

HMBC of Lunagrad B (2) in CD_3OD.

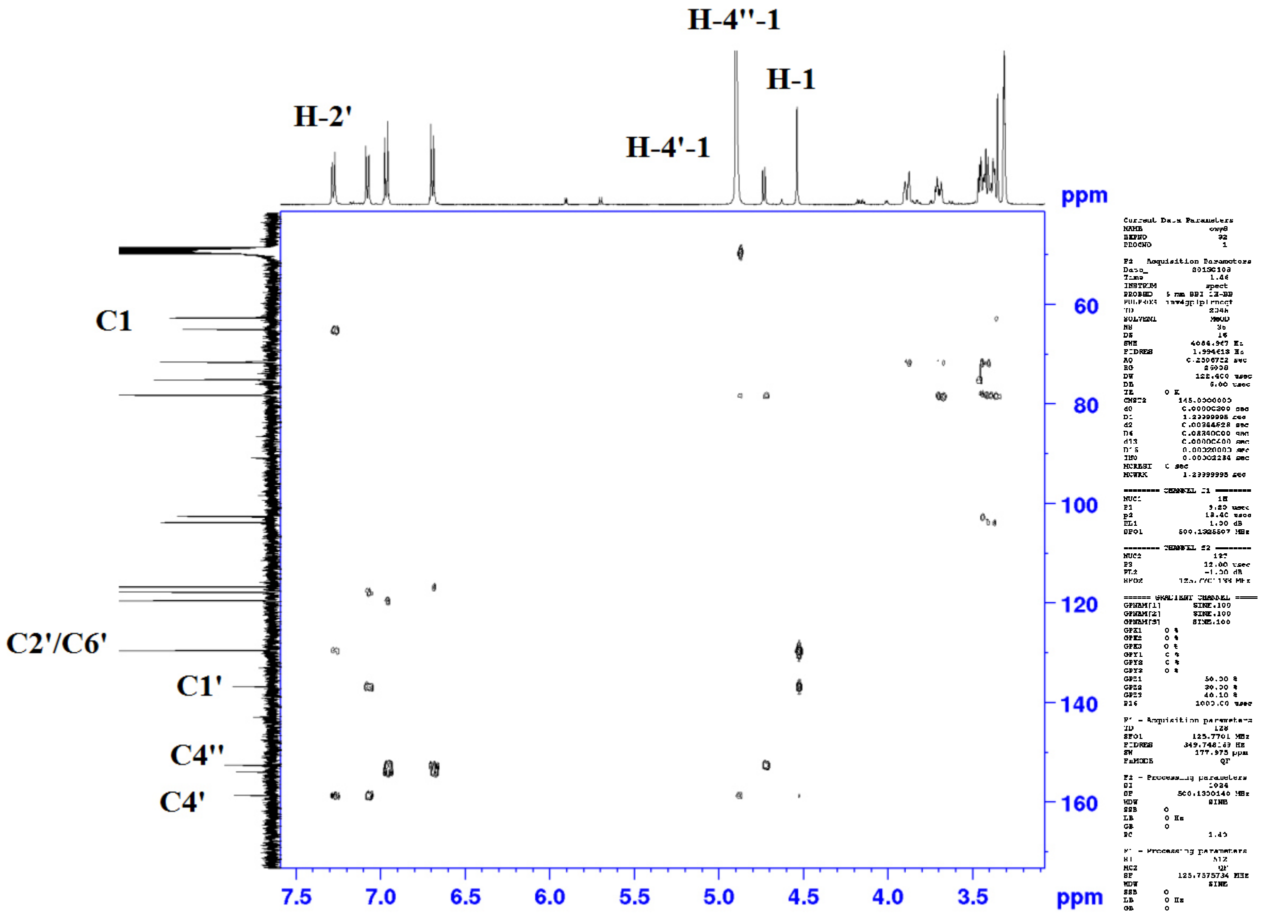

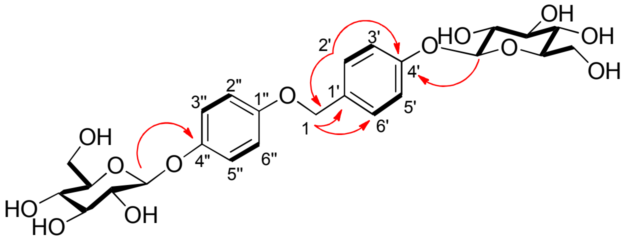

ROESY of Lunagrad B (2) in CD_3OD.

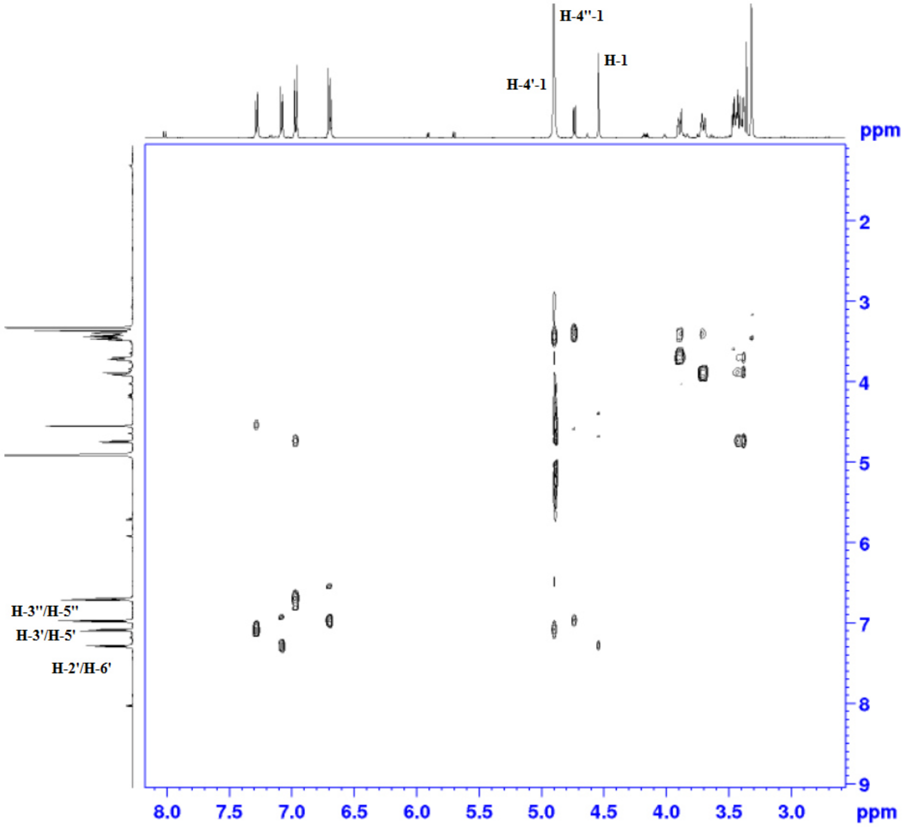


```
Current Data Parameters
NAME                 owy8
EXPNO                  28
PROCNO                  1

F2 - Acquisition Parameters
Date_            20130107
Time                19.17
INSTRUM             spect
PROBHD   5 mm PBI 1H-BB
PULPROG        croesyphpr
TD                   1024
SOLVENT              MeOD
NS                      8
DS                      4
SWH              4006.410 Hz
FIDRES           3.912510 Hz
AQ              0.1277952 sec
RG                 456.15
DW                124.800 usec
DE                   6.00 usec
TE         0 K
d0             0.00011325 sec
D1             1.00000000 sec
d11            0.03000000 sec
d12            0.00002000 sec
d13            0.00000400 sec
IN0            0.00024994 sec
MCREST     0 sec
MCWRK          0.00999999 sec
ST1CNT                  0

-------- CHANNEL f1 --------
NUC1                   1H
P1                   9.20 usec
P15            1200000.00 usec
PL1                 -1.00 dB
PL9                 68.00 dB
PL11                22.00 dB
SFO1          500.1324596 MHz

F1 - Acquisition parameters
TD                    133
SFO1              500.132 MHz
FIDRES          60.165417 Hz
SW                  8.000 ppm
FnMODE        States-TPPI

F2 - Processing parameters
SI                   1024
SF            500.1300084 MHz
WDW                 QSINE
SSB                     2
LB         0 Hz
GB         0
PC                   1.00

F1 - Processing parameters
SI                   1024
MC2           States-TPPI
SF            500.1295536 MHz
WDW                 QSINE
SSB                     2
LB         0 Hz
GB         0
```

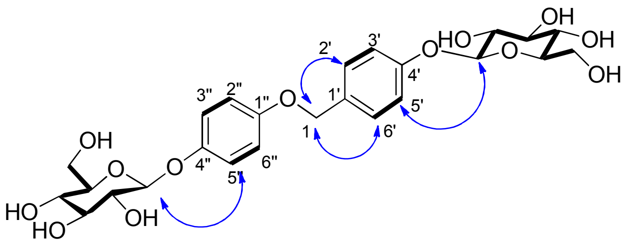

Supplement: Supplementary file 1 [file molecules-23-00099-s001.pdf]
